# Integrated physiological, transcriptomic, and metabolomic analyses of *Chrysanthemum* ‘Boju’ under excessive indole-3-acetic acid stress

**DOI:** 10.3389/fpls.2025.1531585

**Published:** 2025-04-25

**Authors:** Yuqing Wang, Yingying Duan, Na Chen, Wanyue Ding, Yaowu Liu, Shihai Xing

**Affiliations:** ^1^ College of Pharmacy, Anhui University of Chinese Medicine, Hefei, China; ^2^ Joint Research Center for Chinese Herbal Medicine of Anhui of Institute of Herbal Medicine (IHM), Bozhou Vocational and Technical College, Bozhou, China; ^3^ Institute of Traditional Chinese Medicine Resources Protection and Development, Anhui Academy of Chinese Medicine, Hefei, China; ^4^ Joint Research Center for Chinese Herbal Medicine of Anhui of Institute of Herbal Medicine (IHM), Anhui University of Chinese Medicine, Hefei, China; ^5^ Ministry of Education (MOE)-Anhui Joint Collaborative Innovation Center for Quality Improvement of Anhui Genuine Chinese Medicinal Materials, Hefei, China; ^6^ Anhui Province Key Laboratory of Research & Development of Chinese Medicine, Hefei, China

**Keywords:** indole-3-acetic acid, oxidative stress, chrysanthemum morifolium, transcriptomics, metabolomics, plant growth regulation

## Abstract

**Introduction:**

Indole-3-acetic acid (IAA) is a key plant hormone involved in regulating development and responses to abiotic stress. However, excessive IAA treatment can induce oxidative stress, impair growth, and potentially lead to plant death. This study investigates the effects of excessive IAA exposure on the growth of *Chrysanthemum morifolium* (Boju), focusing on the underlying molecular mechanisms.

**Methods:**

We treated *C. morifolium* with 10 mg/L IAA for nine consecutive days. The impact of this treatment was assessed from various perspectives, including physiological (chlorophyll, carotenoids, and MDA content), biochemical (antioxidant enzyme activities), and molecular (transcriptomic and metabolomic analyses).

**Results:**

IAA treatment significantly increased chlorophyll a, chlorophyll b, and carotenoid levels by 37%, 46%, and 25%, respectively, compared to pre-treatment levels, suggesting that *C. morifolium* was experiencing stress. Additionally, the malondialdehyde (MDA) content was 1.79 times higher than pre-treatment levels, confirming oxidative stress. To combat this, the plant enhanced its antioxidant defense mechanisms, as shown by a 93.8% increase in peroxidase (POD) activity and a 45% increase in superoxide dismutase (SOD) activity. Exogenous IAA treatment also led to a significant reduction in endogenous hormone levels, including gibberellins (GA_3_ and GA_4_), abscisic acid (ABA), and IAA, with decreases of 93%, 45%, 99%, and 99%, respectively.Transcriptomic and metabolomic analyses identified 263 differentially expressed metabolites and 144 differentially expressed genes.

**Discussion:**

These results suggest that *C. morifolium* is experiencing stress under prolonged IAA treatment and likely limits its growth by reducing endogenous hormone levels to mitigate oxidative stress. The transcriptomic and metabolomic results showed the upregulation of stress-related genes, including proB (Glutamate 5-kinase), proA (Glutamate-5-semialdehyde dehydrogenase), GAD (Glutamate decarboxylase), and peroxidases, alongside the downregulation of PK (Pyruvate kinase), indicateing a complex response involving the regulation of amino acid biosynthesis, coumaric acid metabolism, starch and sucrose metabolism, and pyruvate metabolism. This study highlights the nonlinear effects of IAA on plant growth and stress responses, emphasizing the intricate molecular mechanisms involved in coping with excessive IAA-induced stress.

## 1 Introduction

*Chrysanthemum morifolium Ramat* can be divided into four strains: Hangju (*C. morifolium* var. Hangju), Boju (*C. morifolium* var. Boju), Chuju (*C. morifolium* var. Chuju), and HuaijuHuaiju (*C. morifolium* var. Huaiju) (National Pharmacopoeia Committee, 2020). Among these, Boju is considered a superior variety of chrysanthemum. In 2014, it was registered as a geographical indication product by the Ministry of Agriculture of China. According to Zhongyao Dacidian, “White chrysanthemum, produced in Bozhou, Anhui, is known as Boju and is considered the highest quality” (Nanjing University of Chinese Medicine, 2006). Traditionally, it has been used in Chinese medicine for its wind-dispersing, heat-clearing, and heat-relieving properties, making it a preferred variety of chrysanthemum in herbal medicine. To further refine the classification of Boju, Liu et al. (2008) distinguished three cultivation types based on their physical characteristics: large Boju, small Boju, and special Boju.

Auxin, the first identified plant hormone, plays a critical role in the regulation of various plant growth and developmental processes. Particularly prevalent among its various forms is IAA(Indole-3-acetic acid), synthesized primarily from tryptophan and non-tryptophan precursors as well as the hydrolysis of IAA conjugates (Casanova-Sáez et al., 2021; Laird et al., 2020). Notably, it promotes plant growth. At the cellular level, auxin stimulates cell division and differentiation; at the plant level, it enhances embryogenesis, and lateral root formation, and regulates photosynthesis processes (Li et al., 2016). However, auxin’s impact on plant growth is dependent on concentration and duration. Low concentrations of auxin foster growth over short durations, while high concentrations can inhibit growth and possibly cause plant mortality if exposure is prolonged. Intriguingly, research reveals that auxin interacts with other plant hormones to form a complex regulatory network (Zhang et al., 2022). It also mediates plant responses to abiotic stresses and pathogens, vital for augmenting stress tolerance (Djami-Tchatchou et al., 2022). Studies show that the application of exogenous IAA can elevate antioxidant enzyme activity in tea plant tissues, enhance photosynthetic efficiency, foster growth, and partially mitigate the adverse effects of drought and heavy metal stress, thus bolstering the plant’s overall resilience (He, 2019). Research by Wang et al. (2023) and Takshak and Agrawal (2017) demonstrate that exogenous IAA can modulate plant physiological and biochemical processes and gene expression across multiple perspectives, fostering plant growth and boosting stress tolerance (Xu et al., 2023). Similarly, Li et al. (2016) report that exogenous IAA heightens plant resilience by prompting soil enzyme activity and increasing microbial biomass. Additionally, Zhao et al. (2024) found that exogenous IAA not only rectifies growth anomalies caused by gene overexpression but also stabilizes auxin homeostasis amid fluctuating endogenous auxin synthesis to ensure normal plant development.

Furthermore, auxin, a key plant hormone, plays a pivotal role in various developmental processes of chrysanthemums. Tanaka et al. demonstrated that in Murashige and Skoog (MS) medium, a high-concentration combination of IAA and kinetin induces somatic embryogenesis in the ray floret explants of the chrysanthemum cultivar ‘Aboukyu’ through *in vitro* studies (Tanaka et al., 2000). Similarly, Fei et al. analyzed two chrysanthemum cultivars with dehiscent and indehiscent anthers, suggesting that variations in IAA levels are closely linked to anther indehiscence (Fei et al., 2016). Likewise, Wen et al. found that external auxin application boosted the expression of the DgD27 gene, thus adjusting lateral branch development in chrysanthemums (Wen et al., 2016). Furthermore, Yuan et al. modified the red-to-far-red light (R:FR) ratio, observing significant alterations in the expression of genes related to IAA metabolism and signal transduction in chrysanthemum axillary buds, implying IAA’s involvement in R:FR-controlled axillary bud germination (Yuan et al., 2018). These findings collectively highlight the versatile roles of IAA in chrysanthemum growth and development, providing a basis for future comprehensive research.

In summary, current research primarily focuses on the role of IAA in regulatory support of plant growth and stability, enhancing resistance to adverse environmental conditions. However, research remains limited regarding the potential inhibitory effects of excessive IAA applications on plant growth; specifically, its impact on physiological, biochemical, transcriptional, and metabolic processes is underexplored. This study conducts a systematic investigation into the physiological, biochemical, transcriptional, and metabolic changes in Boju under high-concentration exogenous IAA treatment. The aim is to elucidate the effects of excessive IAA on its growth, development, and metabolic pathways. These findings will enrich the understanding of gene expression regulation in Boju and will aid in identifying key genes involved in the IAA response.

## 2 Materials and methods

### 2.1 Experimental materials

The Boju samples were collected from 1-year-old Boju plants in the spring, at the base of Bozhou City Construction Group, Bozhou, Anhui Province, China. Associate Professor Qingshan Yang from the Anhui University of Traditional Chinese Medicine later identified these samples as Boju (Duan et al., 2024). The collected Boju samples were subsequently cultivated in the greenhouse of the herbal garden at the Anhui University of Traditional Chinese Medicine, Anhui Province, China. Here, the cultivation environment was maintained at 25°C during the day and 23°C at night, with natural light. Commercially-purchased flower-specific soil – primarily composed of peat, pine bark, coconut coir, and perlite – was used for cultivation. The Boju specimens used in this study are preserved in the Herbarium of the Anhui University of Traditional Chinese Medicine, with the collection number 20240501. All samples used for physiological, biochemical, transcriptomic, and metabolomic analyses in this research were sourced from the same batch of plants.

### 2.2 Experimental methods

We selected Boju plants with similar growth conditions, dividing them into control and treatment groups comprised of six plants each. The control group remained untreated, while those in the treatment group were sprayed twice daily with a 10 mg/L IAA solution. Three samples were collected from each group, and the physiological indexes of Boju were examined on days 0, 3, 6, and 9 of IAA treatment. This included detecting changes in such physiological indicators as chlorophyll, malondialdehyde, protein, superoxide dismutase, and peroxidase. The methodology, with minor adjustments, was referenced from the textbook by Li (2000). Furthermore, we evaluated the alterations in the content of four endogenous hormones: GA_3_(Gibberellin A_3_), GA_4_(Gibberellin A_4_),ABA(Abscisic acid) and IAAbefore and after the exogenous IAA treatment, as well as the variations in transcription and metabolism.

#### 2.2.1 Determination of MDA (malondialdehyde) content

Fresh leaves of Boju (0.5 g) were homogenized in 2 mL of 0.05 M phosphate buffer (pH 7.8). The resulting mixture was transferred to a centrifuge tube, where an additional 6 mL of buffer was added. The mixture was then centrifuged at 10,000 g for 20 min at 4°C. The supernatant was collected and used as the enzyme solution for subsequent assays.

To measure the malondialdehyde (MDA) content, 2 mL of the enzyme solution was combined with 2 mL of a 0.6% thiobarbituric acid solution, along with 4 mL of 5% trichloroacetic acid (TCA). This mixture was incubated in a boiling water bath for 10 min, subsequently removed, and left to cool. Following the cooling process, the solution underwent centrifugation at a speed of 3,000 g for a 15-min duration. The optical density (OD) of the supernatant was then assessed at wavelengths of 532 nm, 600 nm, and 450 nm. Subsequently, the MDA content was calculated utilizing the formula:


$${\text{C}}_{\text{MDA}}\left(\text{µmol}/\text{L}\right)=6.45\left({\text{OD}}_{532}-{\text{OD}}_{600}\right)-0.56{\text{OD}}_{450}$$

$$\text{M\%}\left(\text{µmol}/\text{g}\right)=\frac{{\text{C}}_{\text{MDA}}\times \text{V}}{\text{m}}$$

#### 2.2.2 Determination of SOD (superoxide dismutase) activity

Each prepared solution was aliquoted into four clear EP(Eppendorf) tubes (**Supplementary Table S1**). Two tubes were designated for the experimental group, while the remaining two acted as controls, with the enzyme solution in the control tubes replaced with distilled water. After thorough mixing, one control tube was shielded to prevent light exposure, while the other tubes, including those in the experimental group, were exposed to 4,000× fluorescent lamps for 10 min, maintaining a reaction temperature between 25–35°C. At the end of the incubation period, the reaction was halted by covering all tubes with a black cloth to obstruct further light exposure. The control tube exposed to light served as the blank, and the OD at 560 nm was measured for each tube. Superoxide dismutase (SOD) activity was then calculated using the subsequent equation:


$$SOD_{1}\left(u/g\right)=\frac{\left(A_{CX}-A_{g}\right)\times V_{T}}{0.5\times A_{CX}\times W\times V_{1}}$$

$$SOD_{2}\left(u/mg\right)=\frac{SOD_{1}}{W}$$

In the formula, SOD_1_ represents total enzyme activity, while SOD_2_ denotes specific enzyme activity. A_cx_ stands for the absorbance of the control tube, and A_g_ signifies the change in absorbance of the sample tube. V_T_ refers to the total volume of the sample solution, V_1_ indicates the volume of the sample used in the assay, and W represents the fresh weight of the sample.

#### 2.2.3 Determination of peroxidase activity

The reaction mixture was prepared by combining phosphate buffer, guaiacol, and hydrogen peroxide in appropriate proportions. For the control tube, 3 mL of this reaction mixture was mixed with 1 mL of phosphate buffer, while the experimental tube contained 3 mL of the reaction mixture and 1 mL of an enzyme solution. The resulting reaction mixture was promptly moved to a cuvette, kick-starting the stopwatch for time tracking. The absorbance at the 470 nm wavelength was gauged at 1-min intervals using a spectrophotometer. Finally, the peroxidase activity was computed using the given formula:


$$U\left[u/(\text{g}\cdot \text{min})\right]=\frac{{\text{ΔA}}_{470}\times {\text{V}}_{\text{T}}}{\text{W}\times {\text{V}}_{\text{r}}\times 0.01\times \text{t}}$$

The above equation represents the change in absorbance throughout the reaction. Here, ΔA470 indicates the change in absorbance during the reaction time, W denotes the fresh weight of the sample, V_T_ refers to the total volume of the sample solution, V_r_ indicates the volume of the enzyme solution used in the assay, and “t” represents the reaction time.

#### 2.2.4 Determination of chlorophyll content

Freshly harvested leaves were placed in a mortar. Then, a small quantity of quartz sand and calcium carbonate powder, accompanied by 2–3 mL of 95% ethanol, were added to the leaves. This concoction was ground into a pulp. Subsequently, an extra 10 mL of ethanol was added, and grinding was continued until the tissue turned white. The mixture was left to stand for 3–5 min. Next, the solution was filtered through a funnel into a 25 mL brown volumetric flask. The mortar, pestle, residue, and filter paper were rinsed with a minimal amount of ethanol, and the rinse solution was filtered into the volumetric flask. Finally, the solution was diluted to 25 mL using ethanol. The resulting solution, transferred from the volumetric flask to a cuvette, had its absorbance measured at wavelengths of 665 nm, 649 nm, and 470 nm, using 95% ethanol as the blank. The computed concentrations of chlorophyll a, chlorophyll b, and carotenoids were calculated using the given equations.


$$C_{a}=13.95D_{665}-6.88D_{649}$$

$$C_{b}=24.96D_{649}-7.32D_{665}$$

$$C_{x}=\frac{\left(1000D_{470}-2.05C_{a}-114C_{b}\right)}{245}$$

#### 2.2.5 Determination of soluble protein content

Grind 0.5 g of fresh Boju leaves into a homogenate with 5 mL of distilled water, then centrifuge at 10,000 g for 10 min. Collect 1.0 mL of the supernatant and add 5 mL of Caulmers Brilliant Blue G-250 solution into a test tube; mix thoroughly. After 2 min, measure the absorbance at 595 nm and determine the protein content using the standard curve. The soluble protein content can be calculated using the given equation. In this formula, C represents the calculated protein content value obtained from the standard curve, VT is the total volume of the extracted solution, WF is the fresh weight of the sample, and VS is the volume of the sample spiked during the determination.


$$\text{W}=\frac{\text{C}\times {\text{V}}_{\text{T}}}{{\text{V}}_{\text{S}}\times {\text{W}}_{\text{F}}\times 1000}\left(\text{mg}/\text{g}\right)$$

#### 2.2.6 Determination of plant hormone content

Fresh Boju samples were thoroughly ground in liquid nitrogen. These ground samples were accurately measured and placed into a test tube, followed by the addition of 10 mL of acetonitrile and 8 μL of the internal standard solution. The mixture was then extracted overnight at 4°C, before being centrifuged at 12,000 g for 5 min at a similar temperature. The supernatant was gathered, and 5 mL of acetonitrile was added to the remaining residue. This extraction process was performed twice more. Supplied supernatants were combined, and a suitable quantity of C18 and GCB(graphitized carbon black) was added to purify the extract. The mixture was subsequently centrifuged at 12,000 g for 5 min at 4°C, and then the supernatant was collected. The solvent underwent evaporation under nitrogen, and the leftover residue was re-dissolved in 400 μL of methanol. Before the hormone content analysis using HPLC-MS/MS(High-performance liquid chromatography-tandem mass spectrometry), the solution was filtered through a 0.22 μm organic-phase filter membrane.

The chromatographic conditions were as follows: A Poroshell 120 SB-C18 reversed-phase column was used (2.1 × 150 mm, 2.7 μm) with a column temperature of 30°C; the mobile phase was a ratio of A to B = (methanol/0.1% formic acid): (water/0.1% formic acid). The elution gradient was set as: 0–1 min, 20% A; 1–3 min, 20–50% A; 3–9 min, 50–80% A; 9–10.5 min, 80% A; 10.5–10.6 min, 80–20% A; and 10.6–13.5 min, 20% A. The changes in the endogenous levels of GA_3_, GA_4_, IAA, and ABA in Boju (Boju) after exogenous IAA treatment were measured.

#### 2.2.7 Metabolomics analysis

The Boju samples, given similar growth conditions, were separated into two groups: control and treatment, each containing six biological replicates. The chrysanthemums in the treatment group were sprayed twice daily with a 10 mg/L IAA solution, while the control group was left untreated. Nine days post-treatment, samples were taken from both groups for metabolomics analysis following the standards stipulated by BGI Genomics Co., Ltd, Shenzhen. Each sample, weighing 50 μg, was soaked in 1.5 mL test tubes containing 800 μL of a pre-cooled extraction solution (methanol: H_2_O ratio of 7: 3, v/v) and 20 μL of internal standard 1 (IS1). Homogenization utilized a braided grinder at 50 Hz for 10 min, which was then followed by sonication in a 4°C water bath for 30 min. After resting for 1 h at −20°C, the extracts underwent centrifugation at 14,000 g for 15 min at 4°C. The supernatant (600 μL) was then filtered through a 0.22 μm membrane, and 20 μL of this filtered solution from each sample was combined with mixed QC(quality control) samples to evaluate the reproducibility and stability of the LC/MS (Liquid chromatography-mass spectrometry)analysis. The filtered solutions and mixed QC samples were transferred to 1.5 mL sample vials for instrumental runs. Metabolites were subsequently separated and detected using the UPLC-MS(Ultra-performance liquid chromatography-mass spectrometry) method.

#### 2.2.8 Transcriptomics analysis

Frozen samples were sent to BGI Genomics Co., Ltd, Shenzhen for transcriptome sequencing. The total RNA(ribonucleic acid) enrichment method was used for treatment, and the desired RNA was obtained after purification. The purified RNA was then fragmented. Random N6 primers were used for reverse transcription, followed by cDNA(complementary DNA) duplex synthesis to form double-stranded DNA. The synthesized double-stranded DNA was end-repaired, phosphorylated at the 5′ end, and a sticky ‘A’ overhang was added at the 3′ end. A bulged adapter with a protruding ‘T’ at the 3′ end was then ligated. The PCR(polymerase chain reaction) product was heat-denatured to obtain single-stranded DNA, which was then circularized using primers to generate a single-stranded circular DNA library for sequencing.

#### 2.2.9 Statistical analysis

The experimental data was analyzed using a one-way analysis of variance and independent samples t-test via SPSS Statistics 26.0 software and illustrated using GraphPad Prism 9. A p-value less than 0.05 indicates a significant difference whereas p greater than 0.05 signifies a non-significant discrepancy. Each treatment included three replicates.

## 3 Results

### 3.1 Effects of IAA treatment on physiological and biochemical characteristics of Boju

#### 3.1.1 Changes in malondialdehyde, antioxidant enzymes, chlorophyll, and soluble protein content

**Figure 1:**
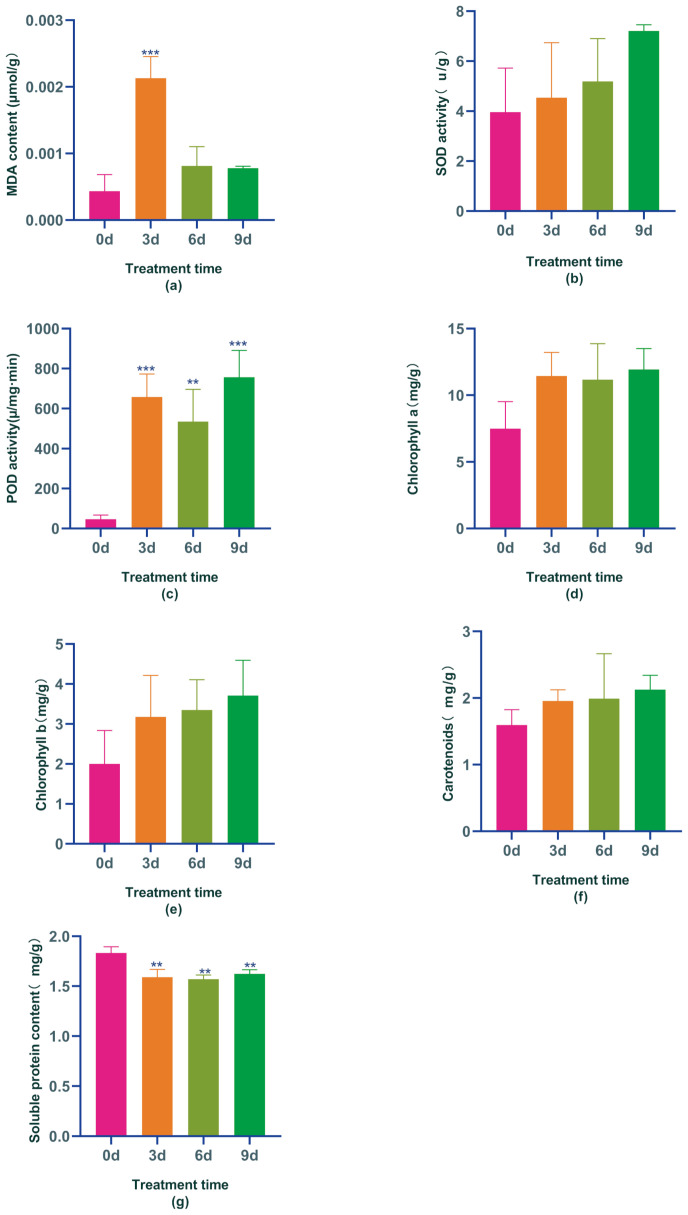
Physiological and biochemical changes in Boju plants from day 0 to day 9 under Indole-3-Acetic Acid(IAA) treatment: **(a)** malondialdehyde content (MDA), **(b)** superoxide dismutase activity (SOD), **(c)** peroxidase activity (POD), **(d)** chlorophyll a content, **(e)** chlorophyll b content, **(f)** carotenoid content, and **(g)** soluble protein content. Error bars represent standard error (SE). Statistical significance is indicated by "**" (P ≤ 0.01) and "***" (P ≤ 0.001).

It has been demonstrated that stress treatment triggers the accumulation of reactive oxygen species (ROS), accelerates membrane lipid peroxidation, and increases malondialdehyde (MDA) content. This study revealed that treatment with IAA significantly raised the MDA content in Boju, which was 4.9, 1.87, and 1.79 times higher after 3, 6, and 9 days of treatment, respectively, compared to day 0. The MDA content stabilized by the ninth day, showing a notable increase on day 3 compared to day 0 (**Figure 1A**). These results suggest that IAA treatment at this concentration leads to the accumulation of ROS and an increase in MDA content in Boju, resulting in cellular damage similar to that caused by abiotic stress.

ROS are toxic compounds that, if not promptly removed, can considerably affect plant growth and development. One key way that plants mitigate ROS effects is by boosting the activity of antioxidant enzymes. The most common ROS scavengers in plants include peroxidase (POD) and superoxide dismutase (SOD). This study revealed that SOD activity in chrysanthemums gradually increased with the application of the hormone IAA, reaching its peak on the 9th day – a 45% increase compared to day 0 (**Figure 1B**). Similarly, POD activities also significantly increased after IAA treatment compared to day 0, showing considerable differences compared to day 0. After 3, 6, and 9 days of IAA treatment, POD activity saw increases of 92.9%, 91.3%, and 93.8%, respectively (**Figure 1C**). These findings suggest that IAA treatment, at this concentration, amplified the activity of antioxidant enzymes and the resilience of Boju, hence preventing lipid membrane peroxidation damage happened due to IAA over-treatment. It ensured a response similar to adversity stress at this time due to IAA over-treatment.

Chlorophyll plays an integral role in plant photosynthesis; higher chlorophyll content indicates stronger photosynthetic activity. However, excessive chlorophyll can produce ROS, leading to cell death. Following IAA treatments, we noted an increase in chlorophyll a, chlorophyll b, and carotenoids in Boju over time. Specifically, the content of chlorophyll a rose by 34%, 32%, and 37% at days 3, 6, and 9, respectively, compared to day 0. Similarly, chlorophyll b content increased by 37%, 40%, and 46% on days 3, 6, and 9, respectively, compared to day 0; carotenoid content also augmented by 18%, 20%, and 25% at days 3, 6, and 9, respectively, compared to day 0 (**Figures 1D-F**). These findings suggest that IAA treatment boosts chlorophyll and carotenoid accumulation in Boju, promoting photosynthesis to help the plant manage the stress induced by excessive IAA. However, the rise in chlorophyll content also resulted in increased ROS production, signaling that Boju was under stress and sustained some damage at this time.

Soluble protein is a crucial substance in osmoregulation. Studies have indicated that soluble protein content typically increases dramatically under stress in plants during normal conditions, yet gradually decreases as stress intensifies. This is because most protein synthesis is inhibited during heightened stress periods, while specific proteins such as aquaporins and late embryogenesis abundant proteins are produced to augment the plant’s resistance to stress. In our research, we observed a decrease in the protein content of Boju leaves following IAA hormone treatment compared to day 0, with all observed changes being significantly different from day 0; decreasing by 15%, 16%, and 13% respectively (**Figure 1G**). This suggests that as the IAA hormone treatment duration extends and stress increases, the synthesis of specific proteins is triggered thereby enhancing the plant’s resilience to the damage induced by excessive IAA treatment at that point in time.

#### 3.1.2 Changes in hormone levels in plants

The current study is designed to investigate the impact of the over-application of exogenous IAA on the growth of Boju plants. Hormones serve as crucial signaling factors enabling plants to respond to stress from adverse conditions. Some research has indicated that the levels of growth hormone (IAA) and gibberellin (GA_3_) in tea tree leaves decrease significantly under drought and high-temperature stress. This suggests that the tea tree slows its growth rate to adapt to these stress conditions by limiting the production of its growth-promoting hormones. Hence, we aim to explore whether the application of exogenous IAA impacts the fluctuation of endogenous hormones in the plant. This could offer a means to gauge the impact of IAA on the plant during such times.

**Figure 2:**
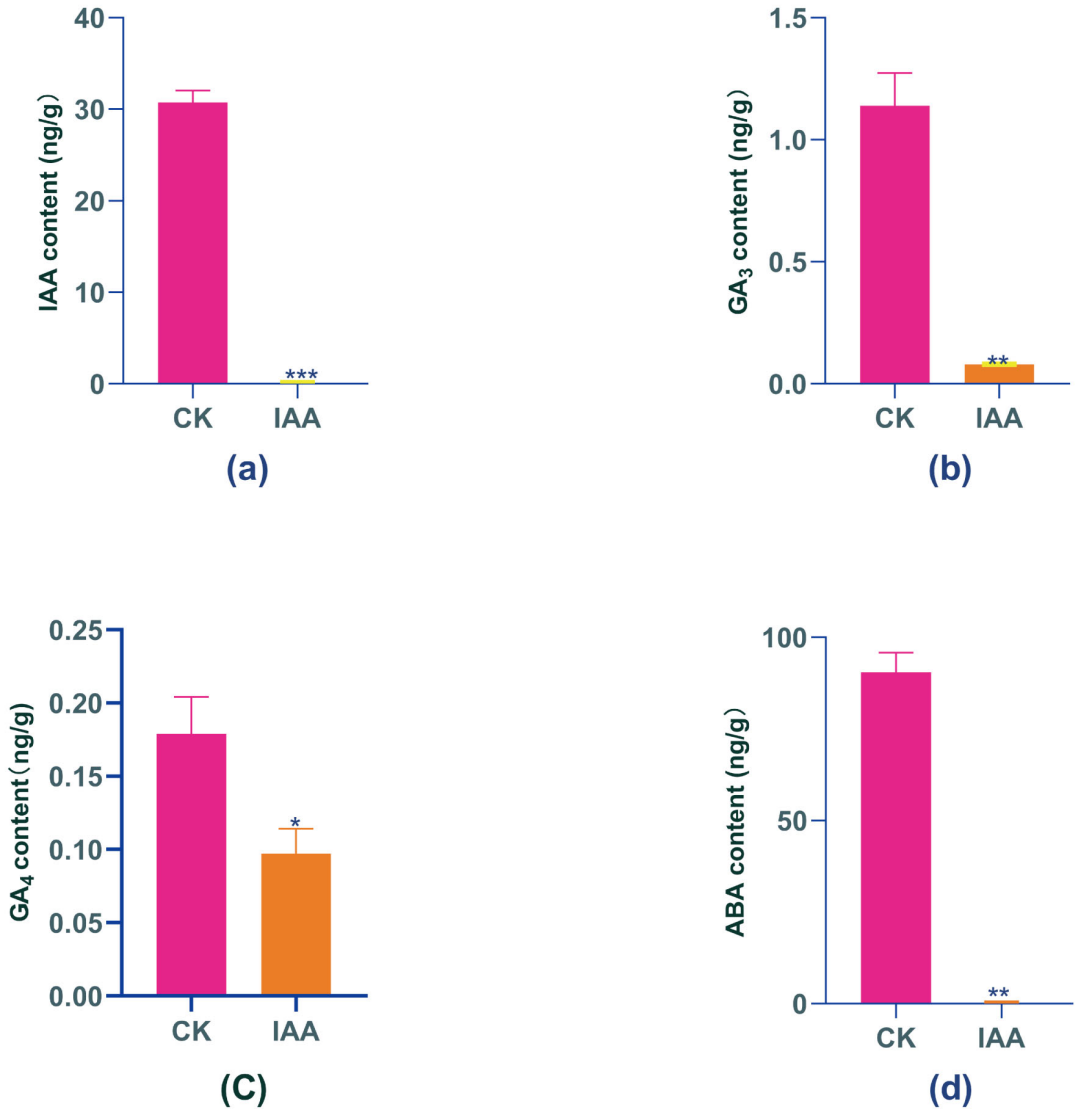
Changes in endogenous phytohormone levels in Boju under exogenous IAA treatment. **(a)** Indole-3-acetic acid (IAA), **(b)** Gibberellin A, (GA,), **(c)** Gibberellin A (GA), and **(d)** Abscisic acid (ABA) content before and after treatment. Error bars represent standard error (SE). Statistical significance is indicated by "*"(P ≤ 0.05), "**" (P<0.01), and "***"(P ≤ 0.001) compared to the control group.

In this study, we predominantly examined the fluctuations in four hormones – endogenous IAA, GA_4_, GA_3_, and ABA – after administering an exogenous IAA hormone. IAA plays a fundamental role as a growth hormone within the plant body, performing a range of functions such as maintaining apical dominance, enabling transport, and facilitating tissue and organ differentiation. It is particularly worth noting that IAA demonstrates dual effects: it promotes growth at low concentrations while inhibiting it at high concentrations Analysis of endogenous IAA in Boju plants before and after the treatment showed a significant 99% decrease in IAA levels by day 9, relative to day 0 (**Figure 2A**).

Gibberellins (GAs) are diterpenoid hormones that regulate various physiological processes, including plant growth, cell elongation, seed germination, and development. GAs also play a role in regulating plant responses to environmental stress. Although 129 gibberellin molecules have been isolated, only a few – including GA_3_ and GA_4_ – are biologically active. Previous studies have demonstrated that IAA positively regulates the expression of GA biosynthesis genes, thereby influencing GA formation. In this study, we observed significant decreases in GA_3_ and GA_4_ levels following IAA treatment. Specifically, GA_3_ decreased by 93% compared to day 0, while GA_4_ decreased by 45% (**Figures 2B, C**).

Thimann et al. proposed that increased levels of abscisic acid (ABA) in plants are a significant cause of leaf senescence, as ABA inhibits protein synthesis and accelerates the degradation of proteins and RNAs in the leaves. Concurrently, ABA enhances plant resistance against abiotic stresses by promoting processes including protein transport, carbon metabolism, and resistance gene expression patterns. In our study, after a 9-day IAA treatment, endogenous ABA levels in Boju decreased by 99% compared to day 0, demonstrating a significant difference from the control (**Figure 2D**). This indicates that while IAA treatment stresses the plants, it does not expedite senescence.

In conclusion, our study determined that all four endogenous hormones – IAA, GA_3_, GA_4_, and ABA – experienced significant reductions in Boju plants following exogenous IAA treatment. This finding suggests that overextended exposure to exogenous IAA does not foster plant growth as expected, but instead incites stress. This stress, in turn, provokes the plant to slow its growth as a response to the hormonal imbalance. Such an adaptive response might encompass a reduction in hormones to alleviate the stress effects induced by the excessive IAA treatment.

### 3.2 Metabolome analysis

#### 3.2.1 Quality control of metabolomics data

The reproducibility of the QC sample assay was used to assess data quality. The primary components included the intensity of the highest ion acquired at each time point, employed to generate the base-peak chromatogram (**Supplementary Figures S1A, B**). Furthermore, the raw mass spectrometry data were processed using MetaX to produce a summary data table. From this table, a detection of 9121 metabolites was confirmed. QC samples were subsequently analyzed, with reproducibility evaluated by the correlation coefficient (R). An R-value closer to 1 indicates fewer systematic errors and superior experimental reproducibility, thus reflecting a higher data quality (**Supplementary Figure S2**). The coefficient of variation (CV) was calculated based on the quantification values of each metabolite in the QC samples, where a lower CV value indicates improved reproducibility of the quantification results (**Supplementary Table S2**).

#### 3.2.2 Analysis of overall metabolites

The analysis of overall metabolites primarily involved qualitative methods, which included demonstrating the detection effects of each sample group through spectrograms and classifying all identified metabolites, followed by counting the number of metabolites in each category. For quantitative analysis, principal component analysis (PCA) plots are utilized to observe contrasting group similarities as a whole and to cluster expression patterns among groups. Furthermore, the number of metabolites detected corresponded with the number of isolated peaks in the base-peak chromatogram (BPC) (**Supplementary Figure S3**). From the data summary table, a total of 9,121 metabolites were counted, out of which 2,308 were identified. Metabolites with classification information in the Kyoto Encyclopedia of Genes and Genomes (KEGG) and HMDB(Human Metabolome Database) databases were categorized into 33 groups (**Supplementary Figure S4**), and the identified metabolites were counted based on the broad categories of the 22 KEGG metabolic pathways they were involved in. Metabolites were predominantly found in lipids, terpenoids, alkaloids, and flavonoids, as well as in the amino acid and carbohydrate metabolic pathways (**Supplementary Figure S5**).

#### 3.2.3 Differential metabolite screening

**Figure 3:**
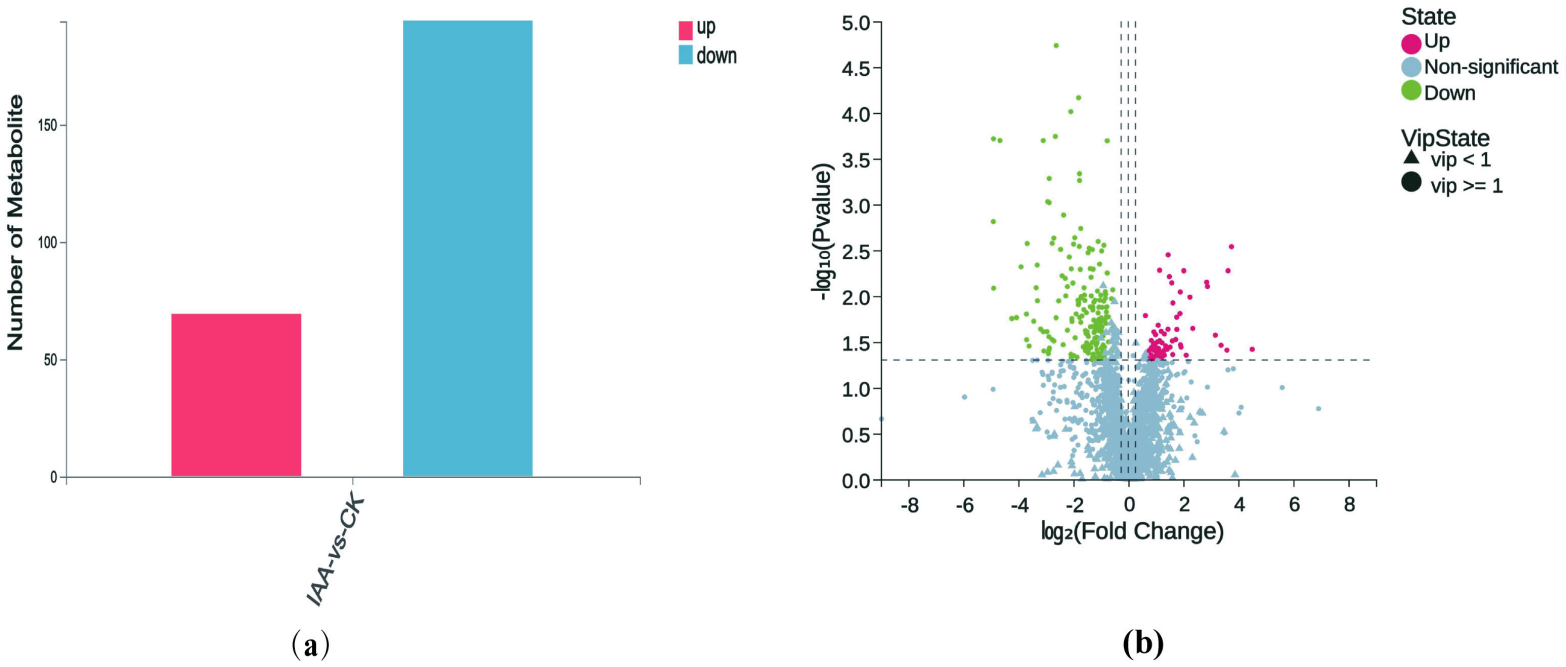
Statistical analysis of differential metabolites. **(a)** Statistical plot of differential metabolites with variable importance in projection (VIP) ≥ 1, fold change ≥ 1.2 or ≤ 0.83, and P-value < 0.05. **(b)** Volcano plot of differential metabolites. The x-axis represents the log-transformed fold change, while the y-axis represents the -log,,-transformed p-value, indicating the statistical significance of changes in metabolite levels. Green dots represent downregulated metabolites, while red dots represent upregulated metabolites. Circles indicate metabolites with VIP ≥ 1, and triangles indicate metabolites with VIP < 1. Non-significant metabolites are shown in gray.

Univariate and multivariate analyses were conducted to detect differential metabolites between the two groups. Initially, we investigated the overall differences using PCA (**Supplementary Figure S6**) and Partial Least Squares Discriminant Analysis (PLS-DA) (**Supplementary Figure S7**). We then validated the OPLS-DA(Orthogonal Partial Least Squares Discriminant Analysis) model (**Supplementary Figure S8**) using a 200-response permutation test. The results suggested a well-fitted model when the red dashed line tilted upwards and the intercept between Q2 and the vertical axis was less than 0 (**Supplementary Figure S9**). Subsequently, we screened differential metabolites based on the following criteria: 1) VIP(variable importance in projection) ≥ 1 in the OPLS-DA model; 2) Fold change ≥ 1.2 or ≤ 0.83; 3) p-value< 0.05 in univariate analysis. We identified a total of 263 differential metabolites, with 69 being up-regulated and 194 down-regulated (**Figure 3A**). A volcano plot of these differential metabolites is illustrated in **Figure 3B**, wherein down-regulated significant metabolites appear in green, up-regulated significant ones in red, metabolites with VIP ≥ 1 as circles, those with VIP< 1 as triangles, and non-significant metabolites in gray.

#### 3.2.4 Differential metabolite analysis of single comparison groups

After screening for differential metabolites, examining the expression patterns and biological functions of these metabolites is crucial. In this study, we analyzed expression patterns through clustering of differential metabolite expression, correlation clustering analysis, and network analysis. The biological functions of these metabolites were scrutinized using pathway annotation and enrichment analysis. We also conducted a comparison analysis of the differential metabolites in individual groups. In addition, we assessed the potential of differential metabolites as markers for growth.

##### 3.2.4.1 Cluster analysis and correlation analysis of differential metabolites

**Figure 4:**
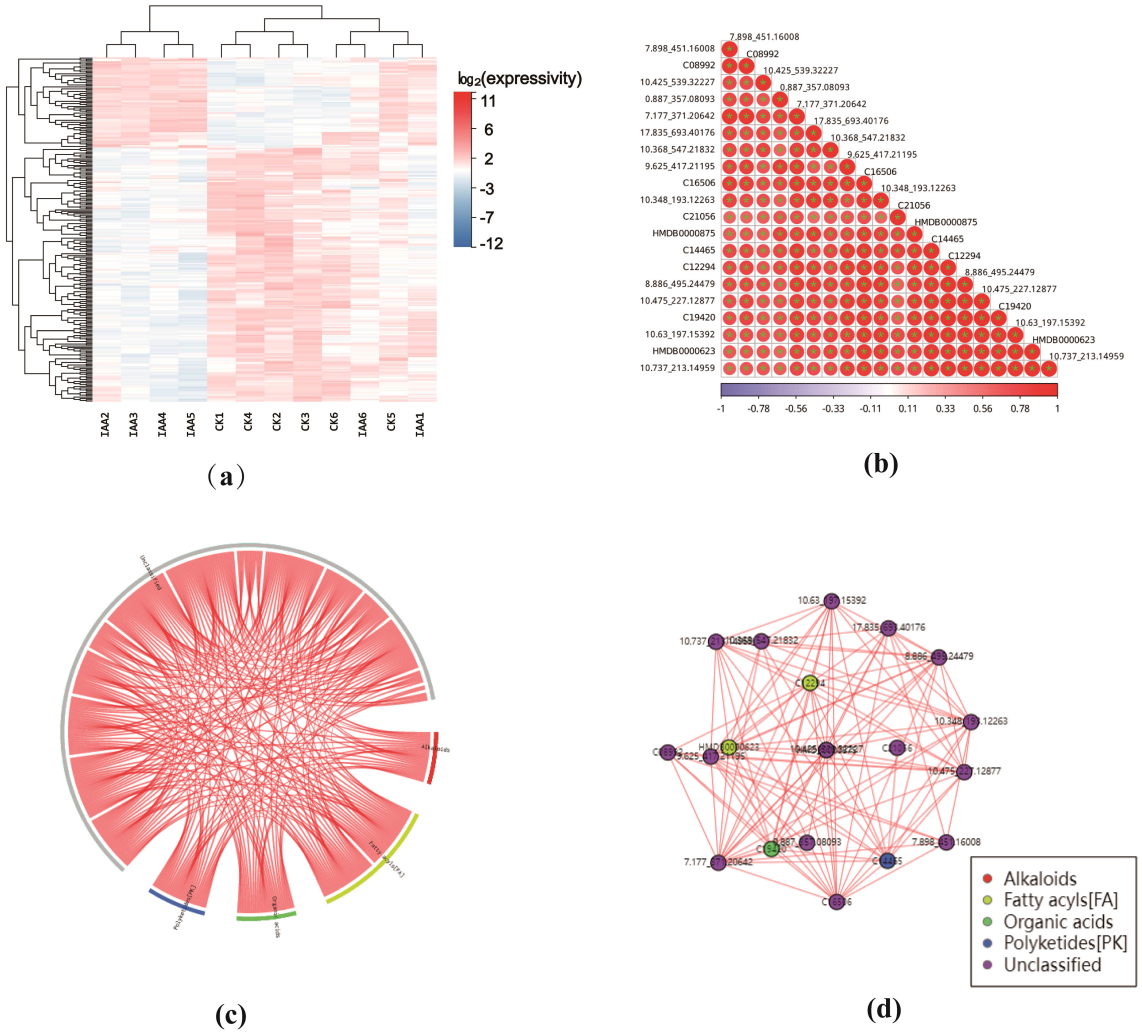
**(a)** Differential Metabolite Heat Map: Each row represents a differentially expressed metabolite, and each column represents a sample. The color intensity indicates the expression level, with blue to red corresponding to low to high expression levels. **(b)** Correlation Heat Map of Differential Metabolites: The correlation heat map was created using the top 20 differential metabolites with the lowest P-values. The darker the color, the larger the absolute value of the correlation coefficient between samples. An asterisk (''*") indicates a P-value < 0.05 for the statistical test of the correlation coefficient. **(c)** Correlation Chord Diagram of Differential Metabolites: In this diagram, the inner circle represents each differentially ex-pressed metabolite, and the arcs on the outer circle represent the categories of these metabolites. The red lines indicate positive correlations between metabolites. **(d)** Correlation Network Diagram of Differential Metabolites: This network diagram shows the correlation relationships between differential metabolites. The red lines represent posi tive correlations (P < 0.05).

The expression of differential metabolites was assessed through cluster analysis, visualizing the pattern of expression in two groups of samples (**Figure 4A**). Data were normalized by the Z-score(standard score), a measure used to evaluate the relative high and low levels of metabolites at the same level (**Supplementary Figure S10**). We probed the correlation between individual metabolites by calculating the Spearman correlation coefficients for all metabolites, subsequently producing a correlation heatmap based on the top 20 differential metabolites with the smallest p-values. Herein, red signifies a positive correlation, while blue indicates a negative correlation. Darker colors represent larger absolute values of the correlation coefficients between samples (**Figure 4B**).

A correlation chord diagram was created **(Figure 4C**) to illustrate the strength of correlations among various metabolite categories. Each link’s starting point in the inner circle represents a significantly distinct metabolite, while the outer circle’s arcs correspond to each metabolite’s classification. The colored lines represent correlations within each metabolite category, with red lines indicating positive correlations. Subsequently, a correlation network diagram was generated based on metabolite-metabolite relationships with a Spearman’s rank correlation coefficient (|r|) > 0.8 and a p-value< 0.05. This diagram prioritizes metabolites that have more connecting edges, implying that these metabolites may hold more central positions within the network (**Figure 4D**). Red lines in this network diagram signify positive correlations.

##### 3.2.4.2 Metabolic pathway enrichment and ROC(receiver operating characteristic) analysis of differential metabolites

**Figure 5:**
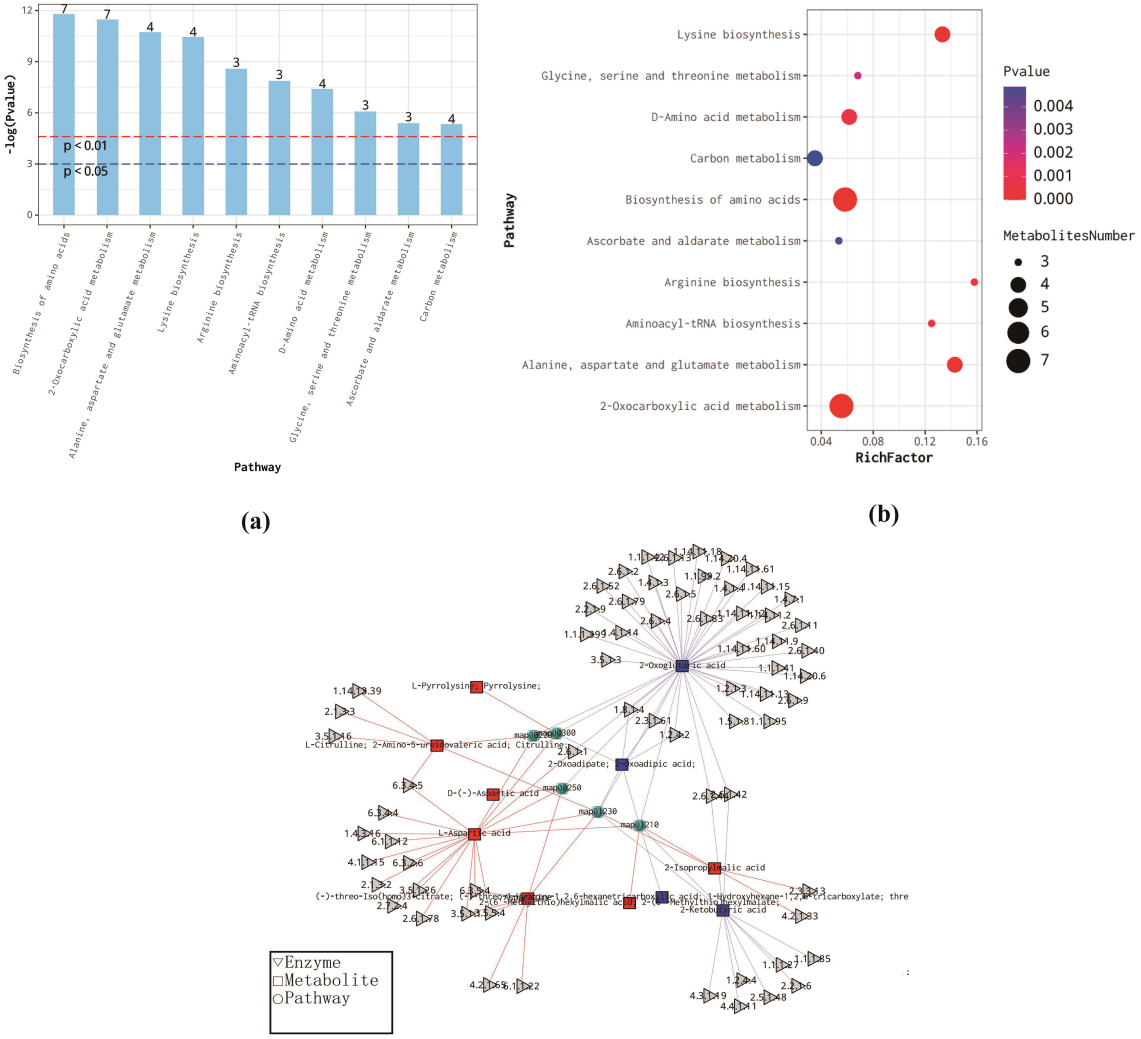
**(a)** Metabolic Pathway Enrichment Analysis Bar Chart **(b)** Bubble Plot of Metabolic Pathway Enrichment Analysis: The x-axis represents the enrichment factor (RichFactor), which is calculated as the n umber of differential metabolites annotated to the pathway divided by the total number of metabolites annotated to the pathway. A higher value indicates a larger proportion of differential metabolites within the pathway. The size of the dots represents the remember of differential metabolites annotated to the pathway. **(c)** Metabolic Pathway Enrichment Analysis Network Diagram: Each dot represents a metabolic pathway, while the connected triangles and squares represent the enzymes and metabolites in that pathway, respectively. Squares represent differential metabolites, with red indicating up-regulated and blue indicating down-regulated metabolites. The gray triangles represent regulatory enzymes associated with the metabolites.

We conducted an enrichment analysis of differential metabolites using the KEGG database and produced bar charts, bubble plots, and network diagrams for the top 10 metabolic pathways with the smallest p-values (**Figures 5A-C**). These visualizations underscore significantly altered metabolic pathways, thereby assisting the interpretation of biological phenotypes.

Based on the enrichment bar and bubble plots, the KEGG pathways of differential metabolites (DEMs) in CK and IAA are primarily enriched in amino acid biosynthesis and metabolism, 2-oxocarboxylic acid metabolism, carbon metabolism, and ascorbic acid and glucuronic acid metabolism. In the enrichment analysis network diagram, each dot signifies a metabolic pathway. The triangles and rectangles connected to these dots represent enzymes and metabolites in that pathway. Squares indicate differential metabolites, with red pointing to up-regulation, blue to down-regulation, and gray triangles representing regulatory enzymes associated with the metabolites. Lastly, the ROC curve analysis of differential metabolites is a standard method for screening potential growth markers, with larger AUC(area under the curve) values indicating that a metabolite may be more suitable for use as a growth marker (**Supplementary Figure S11**).

### 3.3 Transcriptome analysis

#### 3.3.1 Transcriptome sequencing and gene quantitative analysis

Due to marked differences in physiological indicators between the CK and IAA treatments, a differential transcriptome analysis was conducted on these two groups, producing a total of 38.11 Gb of data. Following *de novo* assembly and redundancy removal of clean reads with Trinity, we obtained a total of 334,744 unigenes with a cumulative length of 231,850,720 bp, an average length of 692 bp, an N50 of 1,109 bp, and a GC content of 39.55%. These unigenes were then annotated by comparison with seven functional databases, yielding the following results: 188,725 unigenes in the Non-Redundant Protein Sequence Database (NR, 56.38%), 115,568 in the Nucleotide Sequence Database (NT, 34.52%), 112,100 in Swiss-Prot (33.49%), 94,686 in the Eukaryotic Clusters of Orthologous Groups (KOG, 28.29%), 116,277 in KEGG (34.74%), 149,638 in GO (44.70%), and 86,485 in the Protein Families Database (Pfam, 25.84%) were functionally annotated.Using TransDecoder, 103,247 coding sequences (CDSs) were detected. Additionally, 22,799 simple sequence repeats (SSRs) were identified in 20,082 unigenes, and 3,601 unigenes encoding transcription factors were predicted. PCA was executed on control and treated samples (**Supplementary Figure S12**) to pinpoint differentially expressed genes. The dispersion of gene expression levels across samples was evaluated using box plots (**Supplementary Figure S13**), and Venn diagrams were created for genes in treatment and control groups (**Supplementary Figure S14**). Interestingly, a total of 206,098 genes were expressed in the treatment and control groups.

#### 3.3.2 Differentially expressed genes

**Figure 6:**
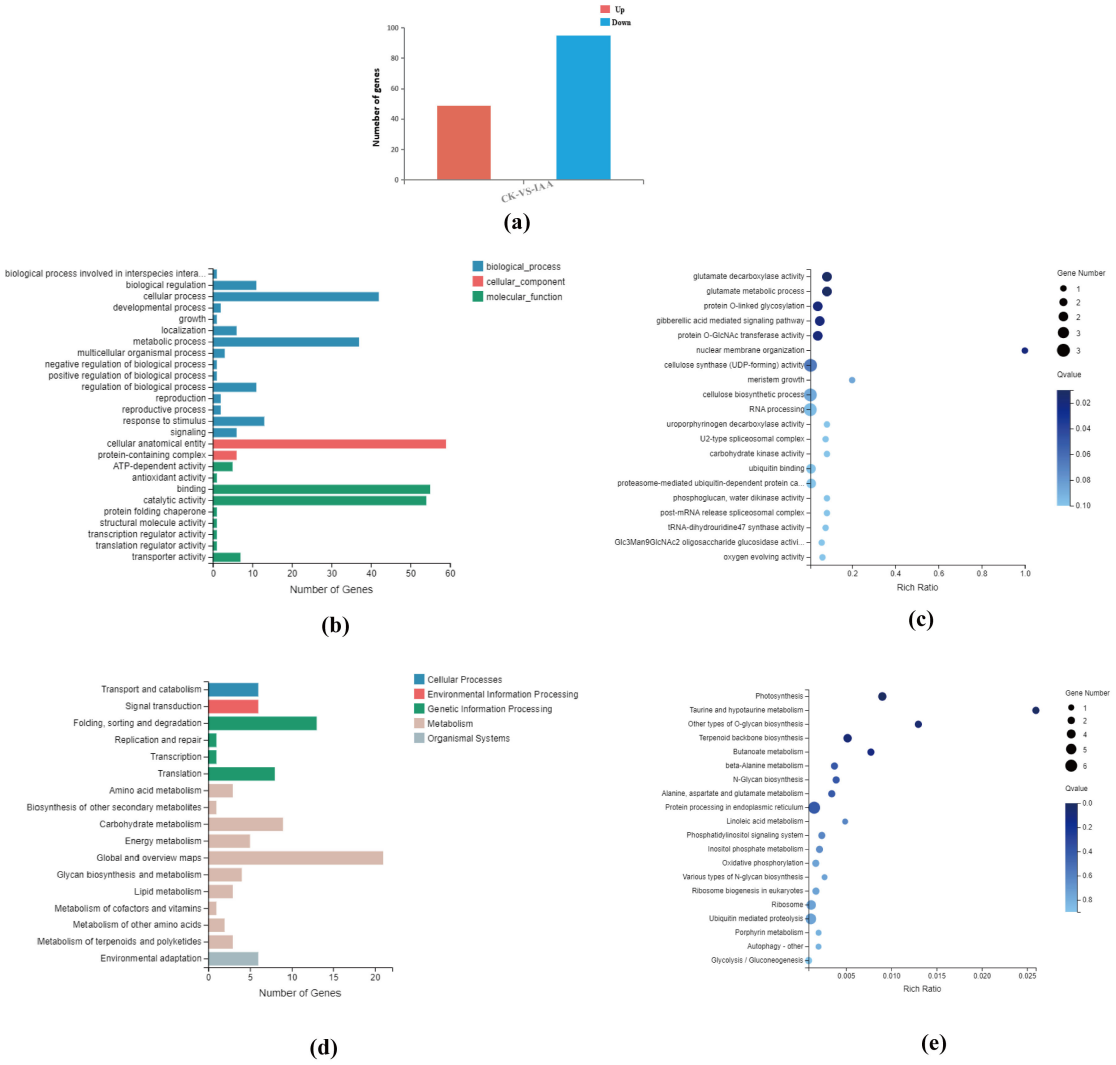
**(a)** Statistical map showing the number of differential genes identified in the analysis. **(b)** Classification of differential genes based on Gene Ontology (GO) categories, representing their biological processes, molecular functions, and cellular components. **(c)** GO enrichment bubble map of differential genes, highlighting the most significantly enriched GO terms (P<0.05). **(d)** Classification of differential genes into Kyoto Encyclopedia of Genes and Genomes (KEGG) pathways, illustrating the associated biological pathways. **(e)** KEGG pathway enrichment bubble map for differential genes, displaying the most significantly enriched pathways (P<0.05).

Intergroup difference analysis was conducted using Poisson Distribution, with the thresholds being a Fold Change ≥ 2 and an FDR(False Discovery Rate) ≤ 0.001. A total of 144 differential genes were identified, with 49 up-regulated and 95 down-regulated (**Figure 6A**). Subsequently, based on GO and KEGG annotation results, these differential genes underwent further functional classification and enrichment analysis.

In the GO analysis, results were categorized into three functional groups: molecular function, cellular component, and biological process (**Figure 6B**). Functional categories that displayed a Q value ≤ 0.05 were deemed significantly enriched. This analysis exposed that the differential genes were significantly enriched in 183 GO terms. The top 20 GO terms with the smallest Q values were chosen and visualized as an enriched GO bubble plot. The plot reveals significant enrichment in glutamate decarboxylase activity, glutamate metabolic process, protein O-linked glycosylation, gibberellic acid-mediated signaling pathway, and protein O-GlcNAc transferase activity (**Figure 6C**).

Moreover, KEGG pathway analysis identified that differential genes partook in 51 metabolic pathways. These pathways were sorted into seven branches, with the differential genes in this study mostly involved in five major branches: Cellular Processes, Environmental Information Processing, Genetic Information Processing, Metabolism, and Organismal Systems (**Figure 6D**).

Based on the KEGG pathway annotation classification, p-values were calculated, and pathways with a Q value ≤ 0.05 were deemed significantly enriched. We identified 43 KEGG pathways significantly enriched in the differentially expressed genes found in this study. The top 20 pathways with the smallest Q values were selected for the KEGG enrichment bubble plot. The analysis indicated that the differentially expressed genes were mainly enriched in pathways associated with photosynthesis, taurine, and hypotaurine metabolism, other types of O-glycan biosynthesis, and terpenoid backbone biosynthesis. Other enriched pathways included butanoate metabolism, beta-alanine metabolism, N-glycan biosynthesis, alanine, aspartate, and glutamate metabolism, among others (**Figure 6E**).

### 3.4 Joint analysis of transcriptome and metabolome

**Figure 7:**
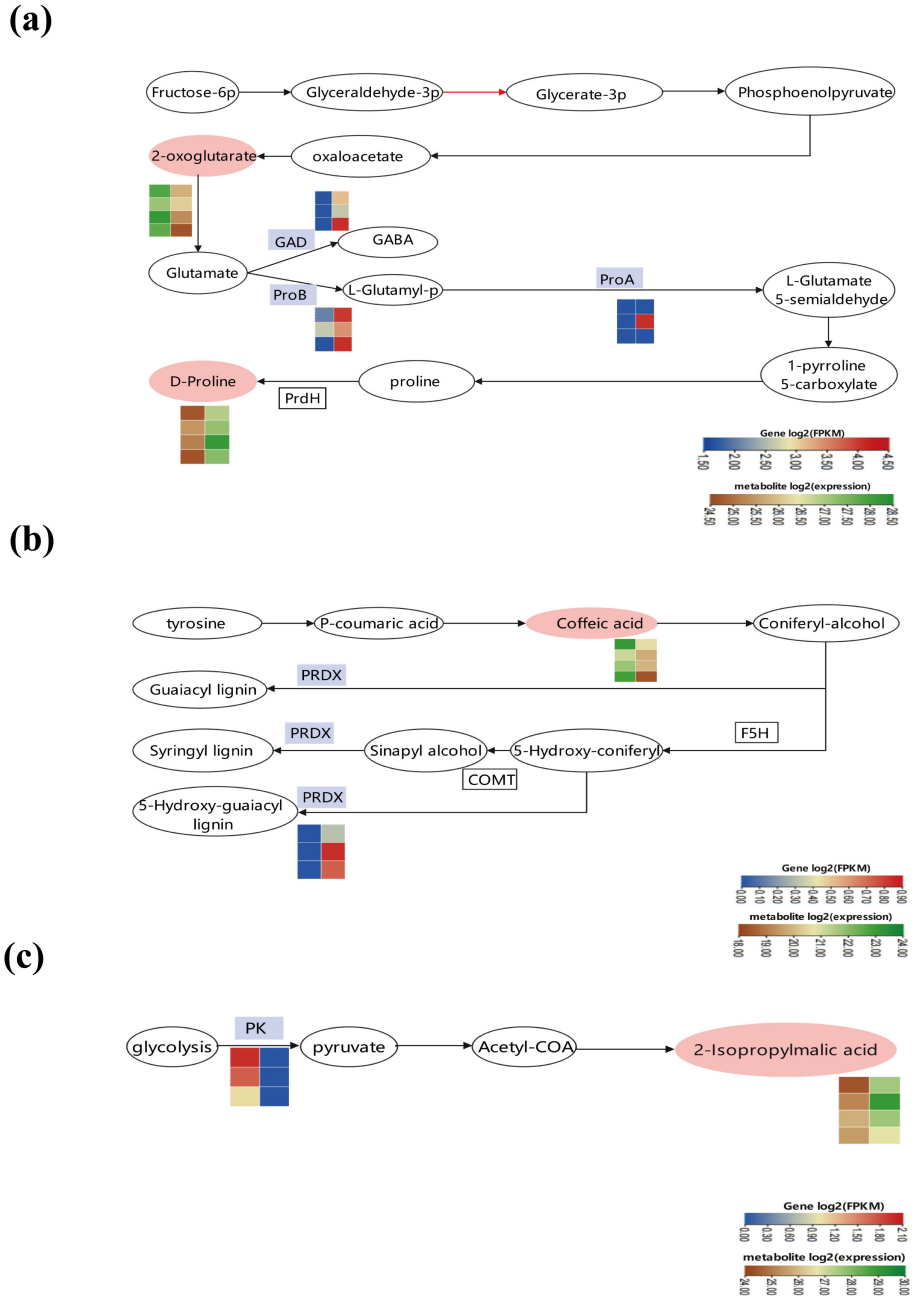
Changes in major metabolic pathways in Boju plants after 9 days of Indole-3-Acetic Acid (IAA) treatment. Differentially expressed genes and metabolites involved under IAA treatment in **(a)** amino acid biosynthesis and metabolism, **(b)** phenylpropanoid biosynthesis, and **(c)** pyruvate metabolism. Pink ovals represent differentially expressed metabolites (DEMs) with altered content, while white ovals represent DEMS with unchanged content. Blue boxes indicate enzymes encoded by differentially expressed genes (DEGs). The color of the rectangles indicates the changes in the expression levels of these DEGs or DEMS under IAA treatment, as shown in the scale bar.

By merging transcriptomics and metabolomics analyses, we discovered that the differential genes and metabolites were primarily concentrated in three key pathways: “amino acid biosynthesis and metabolism,” “phenylpropanoid synthesis,” and “pyruvate metabolism” (**Figure 7**). In the “amino acid biosynthesis” pathway, IAA treatment noticeably boosted amino acid metabolism. In particular, IAA treatment increased the expression of enzymes such as glutamate 5-kinase (ProB), glutamate-5-semialdehyde dehydrogenase (ProA), and glutamate decarboxylase (GAD) compared to the control. Meanwhile, IAA treatment resulted in the reduction of 2-oxoglutarate and the enhancement of D-proline within the “amino acid biosynthesis and metabolism” pathway (**Figure 7A**). In the phenylpropanoid synthesis pathway, there was an up-regulation of peroxidase (PRDX) expression, indicating that IAA treatment stimulated phenylpropanoid biosynthesis to alleviate damage from environmental stress (**Figure 7B**). In the pyruvate metabolism pathway, a decrease in the PK(pyruvate kinase) gene expression facilitated pyruvate metabolism, going hand in hand with an increase in the metabolite 3-carboxy-3-hydroxy-4-methylpentanoate (**Figure 7C**).

## 4 Discussion

Auxin is the earliest discovered plant hormone. With advancements in biotechnology, its synthesis and metabolism are now widely studied and applied to promote plant growth and enhance crop yields (Zhang et al., 2018; Cucinotta et al., 2020; Wang et al., 2019; Chen et al., 2022). However, the growth-promoting effects of auxin are influenced by its concentration and duration of exposure. This aligns with the concentration-dependent effects of IAA reported by Aloni et al. (2006). At low concentrations, IAA promotes organic matter accumulation during plant growth and development, regulating processes such as cell expansion and root development. Within an optimal concentration range (0–0.2 mg/L), IAA enhances cell elongation and division, thereby accelerating plant growth (Butova et al., 2023). However, when IAA exceeds a certain threshold, it exhibits herbicidal effects, inhibiting plant growth and inducing oxidative stress, leading to growth suppression (Gomes and Scortecci, 2021). Moreover, under stress conditions, elevated IAA levels may trigger abnormal gene expression. For instance, aluminum stress has been shown to cause excessive IAA accumulation, inhibiting root elongation (Zhang et al., 2022). Sun et al. (2023) also investigated the effects of phytohormones on the growth and lateral branch development of Cunninghamia lanceolata. Their findings suggest that while low IAA concentrations promoted seedling height, higher concentrations suppressed growth (Guo, 2015).

In our investigation, we treated *C. morifolium* (of the Boju variety) with 10 mg/L IAA via foliar spraying two times daily over nine consecutive days. This treatment model displayed physiological changes pointing toward a stress response. Specifically, IAA treatment corresponded with an increase in MDA content (Morales and Munné-Bosch, 2024; Liang et al., 2019; Lu et al., 2023), the enhancement of the POD and SOD antioxidant enzyme activities (Wang et al., 2024; Nadarajah, 2020; Wei et al., 2020), and an elevation of chlorophyll and carotenoid levels (Hörtensteiner, 2013). However, the soluble protein content indicated a decrease (Jaimes-Miranda and Chávez Montes, 2020; Schwartz et al., 1994). These biochemical adjustments suggest that, at this specific concentration, IAA treatment behaves as a stressor for the Boju variety, provoking oxidative stress. Consequently, the activation of the antioxidant enzyme system improves the plant’s defense mechanisms and boosts its stress tolerance, which aligns with the inhibitory effects that high IAA concentrations have on growth.

This study aims to investigate the effects of excessive exogenous IAA application on the growth of *C. morifolium* (Boju variety). Plant hormones are critical signaling molecules that mediate plant responses to environmental stresses (Yu et al., 2020; Waadt et al., 2022)and participate in various growth processes (Hedden, 2020; Shohat et al., 2021; Ueguchi-Tanaka, 2023; Xie and Chen, 2020; Thimann, 1980). Numerous studies have demonstrated interactions between auxin (IAA) and other hormones in plants (Hu et al., 2022). For instance, under drought and high-temperature stress, the levels of IAA and gibberellin (GA_3_) in tea tree leaves significantly decrease (Tang et al., 2023). This suggests that tea trees adapt to stress conditions by reducing growth-promoting hormones to slow down growth and enhance stress tolerance. Therefore, we analyzed the endogenous levels of gibberellins (GA_3_ and GA_4_), IAA, and ABA in *C. morifolium* following IAA treatment. The results indicated that all four hormone levels decreased, with IAA, GA_3_, and ABA exhibiting significant reductions, while GA_4_ showed a relatively smaller decline. Significant differences were observed between the treated and control groups. This reduction in hormone levels may be attributed to the inhibitory effect of excessive exogenous IAA on plant growth, triggering stress-like responses. Consequently, *C. morifolium* may adapt to this stress condition by downregulating growth-promoting hormones to delay growth.

We discovered that IAA treatment modified hormonal and physiological levels of Boju, replicating stress-like effects. To investigate the alterations in transcription and metabolism, we examined the transcriptome and metabolome of Boju samples before and after IAA treatment. Transcriptome testing illustrated that out of 144 differentially expressed genes, 49 were up-regulated and 95 were down-regulated. These were analyzed and enriched using GO and the KEGG pathway.

In the GO classification, differential genes were segregated into three primary categories: molecular function, cellular component, and biological process. The top 20 GO terms displaying the smallest Q value were enriched in the bubble chart, with significant expression in glutamate decarboxylase activity, glutamate metabolic process, protein O-linked glycosylation, gibberellic acid-mediated signaling pathway, and protein O-GlcNAc transferase activity.

Furthermore, by KEGG annotation, we mapped these differentially expressed genes to 51 metabolic pathways, where they were significantly enriched in 43 KEGG pathways. The top 20 pathways featuring the smallest Q value were exhibited in the enriched KEGG bubble map. Differentially expressed genes were primarily concentrated in pathways such as photosynthesis, taurine, and hypotaurine metabolism, other types of O-glycan biosynthesis, terpenoid backbone biosynthesis, butanoate metabolism, β-alanine metabolism, N-glycan biosynthesis, and alanine, aspartate, and glutamate metabolism.

The metabolomics results revealed a total of 9121 metabolites, of which 2308 were identified. The metabolites with classification information in KEGG and HMDB databases were grouped into 33 categories. The number of identified metabolites was tallied based on their involvement in 22 KEGG metabolic pathways, including lipids, terpenoids, alkaloids, flavonoids, as well as amino acid and carbohydrate metabolic pathways. Univariate and multivariate analyses were conducted to screen for variations in metabolites between the two groups. Univariate results indicated that 263 metabolites were differentiated, with 69 being up-regulated and 194 down-regulated. Multivariate analyses were carried out to statistically evaluate differences between the two groups using PCA, PLS-DA, OPLS-DA (Andries and Vander Heyden, 2021; Wang and Lê Cao, 2023), and permutation tests to aid in the isolation of differential metabolites. The differentiated metabolites were subsequently subjected to multiple variance analyses. Metabolic pathway enrichment analyses showed that the KEGG pathway of DEMs in CK and IAA was significantly enriched in amino acid biosynthesis and metabolism, 2-oxocarboxylic acid metabolism, carbon metabolism, and ascorbic acid and glucuronic acid metabolism.

Based on the results of transcriptomics and metabolomics, we found that the up-regulation of glutamate 5-kinase, glutamate-5-semialdehyde dehydrogenase, and glutamate decarboxylase occurred in amino acid synthesis and metabolism pathways in Boju after IAA treatment. This promotes the metabolism and synthesis of several amino acids. Furthermore, the up-regulation of peroxidase in the phenylpropanoid synthesis pathway fosters the synthesis of various phenylpropanoids. This suggests that IAA hormone treatment activates phenylpropanoid biosynthesis to counteract the damage caused by stress. At the same time, the down-regulation of pyruvate kinase reduces pyruvate synthesis. Pyruvate is a product of glycolysis in cellular metabolism, which links oxidative and catabolic metabolism in cells, occupies a significant place in the cellular metabolic network, and responds to stress (Timón-Gómez et al., 2013). Additionally, our transcriptomic findings showed an up-regulation of sucrose-phosphate synthase, cellulose 1,4-beta-cellobiosidase, beta-amylase, and numerous other sucrose, starch, and cellulose-related synthases during carbon metabolism. This provides energy and carbon skeletons for plant growth. It also suggests that plants could resist damage from excessive external application of IAA by storing high amounts of sugar. Moreover, the up-regulation of genes linked to carbon-fixing enzymes in the carbon metabolism pathway is beneficial for converting solar energy into organic matter stored within the plant, thus enhancing the plant’s resilience under such conditions.

This study reveals that excessive treatment with IAA induces physiological and metabolic responses in plants similar to those triggered by stress. It activates the antioxidant enzyme system, modulates hormone levels, and regulates multiple key metabolic pathways at the transcriptomic and metabolomic levels. Collectively, these changes enhance the stress tolerance of chrysanthemums. The findings provide deep insights into the role of plant hormones in stress responses and offer a theoretical basis for future regulation of plant growth and crop yield improvement.

## 5 Conclusions

This study utilized a multi-omics approach to examine the response mechanisms of Boju after continuous treatment with 10 mg/L IAA over 9 days. This treatment mimicked stress adversity effects. The results demonstrated changes in antioxidant enzymes, malondialdehyde, soluble proteins, and chlorophyll levels in Boju, thereby producing effects akin to stress adversity. Consequently, the plants adapted by modifying various internal indicators, thereby enhancing their resistance.

The exploration also encompassed alterations in endogenous hormones in Boju plants post-exogenous IAA treatment. The levels of endogenous IAA, GA_3_, GA_4_, and ABA significantly decreased post-treatment. This implies that continuous exogenous IAA treatment at 10 mg/L inhibits plant growth, eliciting a stress-like adversity effect. The plants reacted to this stress by reducing various growth hormone levels, thereby slowing growth.

Furthermore, the transcriptome and metabolome showed that plants regulated amino acid biosynthesis, phenylpropanoid synthesis, starch and sucrose metabolism, and pyruvate metabolism amid IAA-induced stress-like effects. It achieved this through the up-regulation of genes such as proB, proA, GAD, peroxidase, and the down-regulation of PK genes. This helped plants to resist the stress-like effect, thus boosting the plant’s stress tolerance.

This study demonstrated that the growth hormone, IAA, does not consistently promote plants. In fact, inappropriate spraying of IAA in the agricultural production process may, paradoxically, inhibit plants, creating effects similar to stress adversity.

## Data Availability

The original contributions presented in the study are included in the article/**Supplementary Material**. Further inquiries can be directed to the corresponding authors.
