# Supplementary material for: Integrated physiological, transcriptomic, and metabolomic analyses of Chrysanthemum ‘Boju’ under excessive indole-3-acetic acid stress
Source: Front Plant Sci. 2025 Apr 25;16:1531585. doi: 10.3389/fpls.2025.1531585 (PMC12061948; doi:10.3389/fpls.2025.1531585)
Supplement: Supplementary file 1 [file DataSheet1.pdf]

Supplementary Material

1 Supplementary Figures and Tables

For more information on Supplementary Material and for details on the different file types accepted, please see here.

1.1 Supplementary Figures

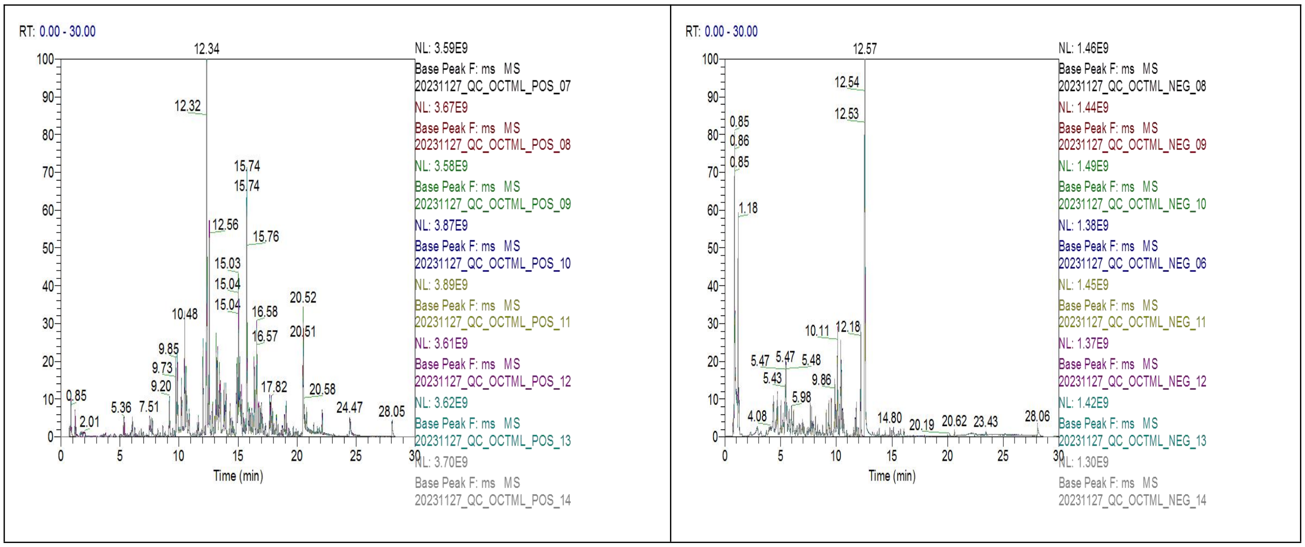

Figure S1. All QC samples in positive (a) and negative ion (b) modes were overlapped. The horizontal coordinate was the retention time, and the vertical coordinate was the response intensity of the strongest ion in the signal collected at this time point.The high overlap of response intensity and retention time of chromatographic peaks indicates that the instrument has good stability and good data quality.

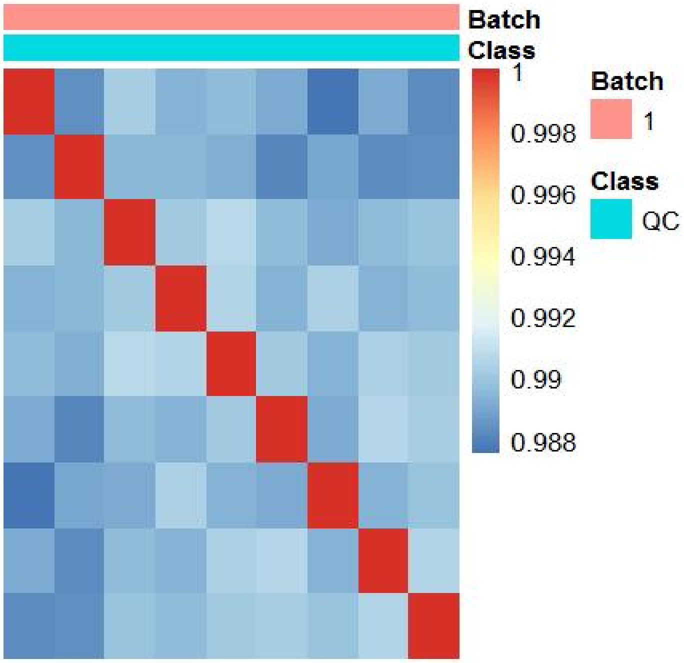

Figure S2. Correlation heat map of QC samples. The higher the correlation of the QC samples (the closer the R is to 1), the lower the systematic error, the better the repeatability of the experiment and the higher the quality of the data.

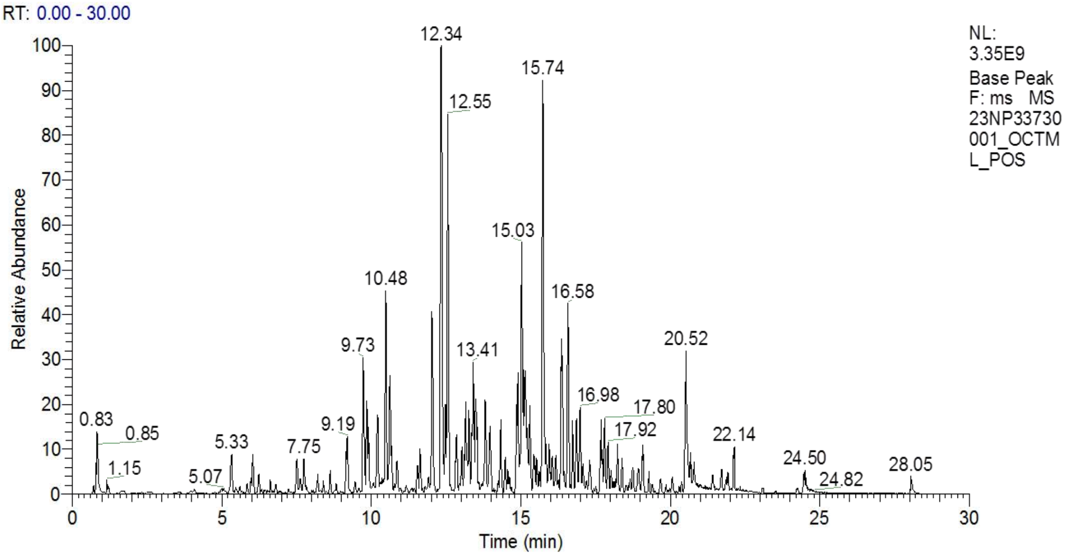

Figure S3.A typical base-peak spectrogram of the sample. The horizontal coordina-te is the retention time, and the vertical coordinate is the response intensity of the strongest detachment of the signal ac quired at that time point.

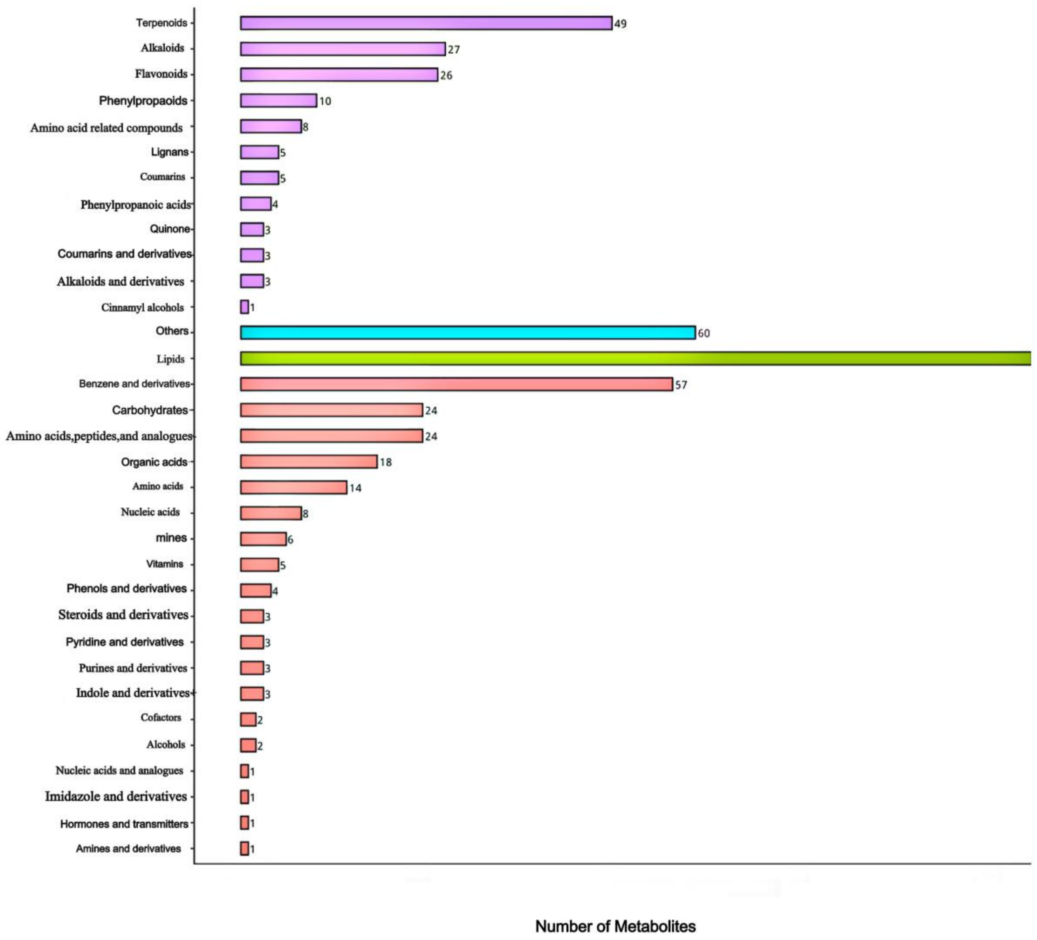

Figure S4. Metabolite classification bar chart. In the figure, the vertical coordinate is the class to which the metabolite belongs, and the horizontal coordinate is the number of metabolites

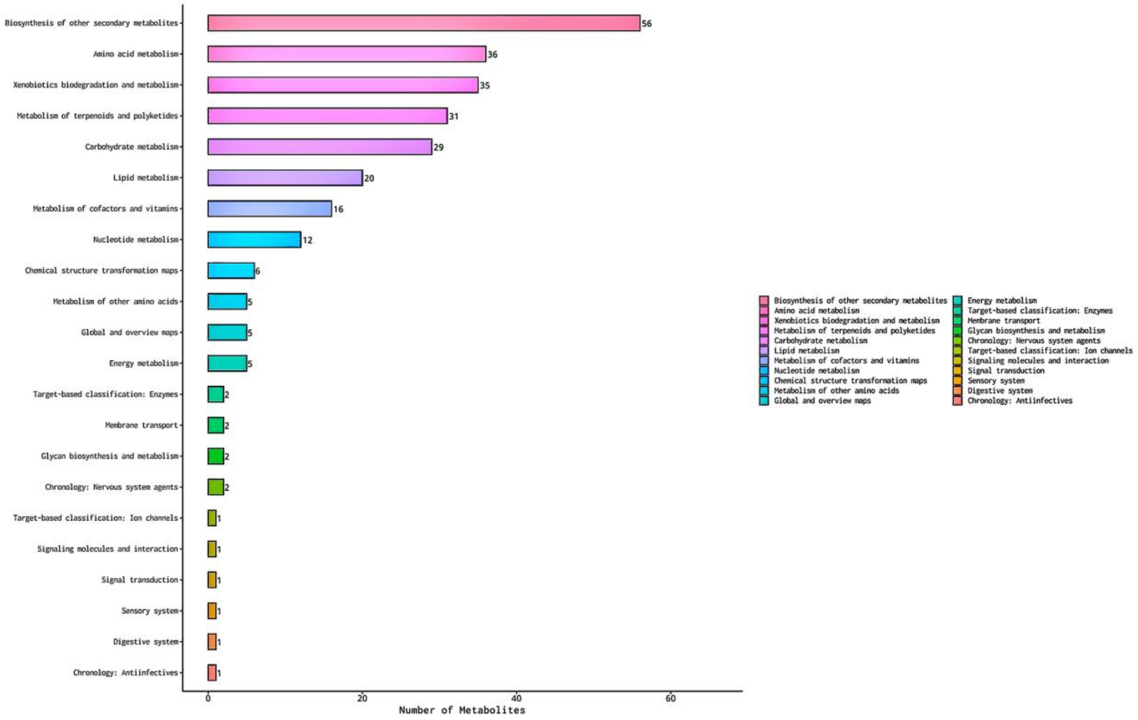

Figure S5. Metabolic pathway classification bar chart. In the figure, the vertical coordinate is the class of metabolic pathway, and the horizontal coordinate is the number of metabolites.

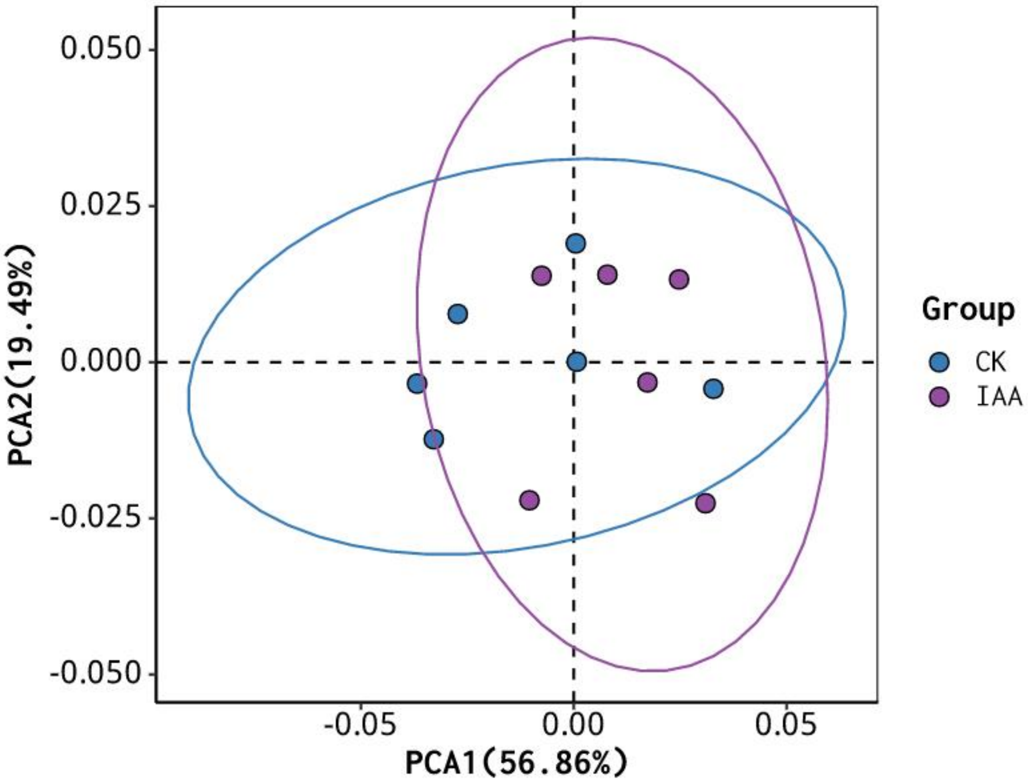

Figure S6. Score chart of PCA analysis model for each group. The horizontal coordinate is the first principal component PCA1, the vertical coordinate is the second principal component PCA2, and the numbers in parentheses are the scores of this principal component, representing the percentage of the total difference explained by the corresponding principal component. In the PCA score plot, each point represents a sample, different groups are marked with different colors, and the ovals are 95% confidence intervals.

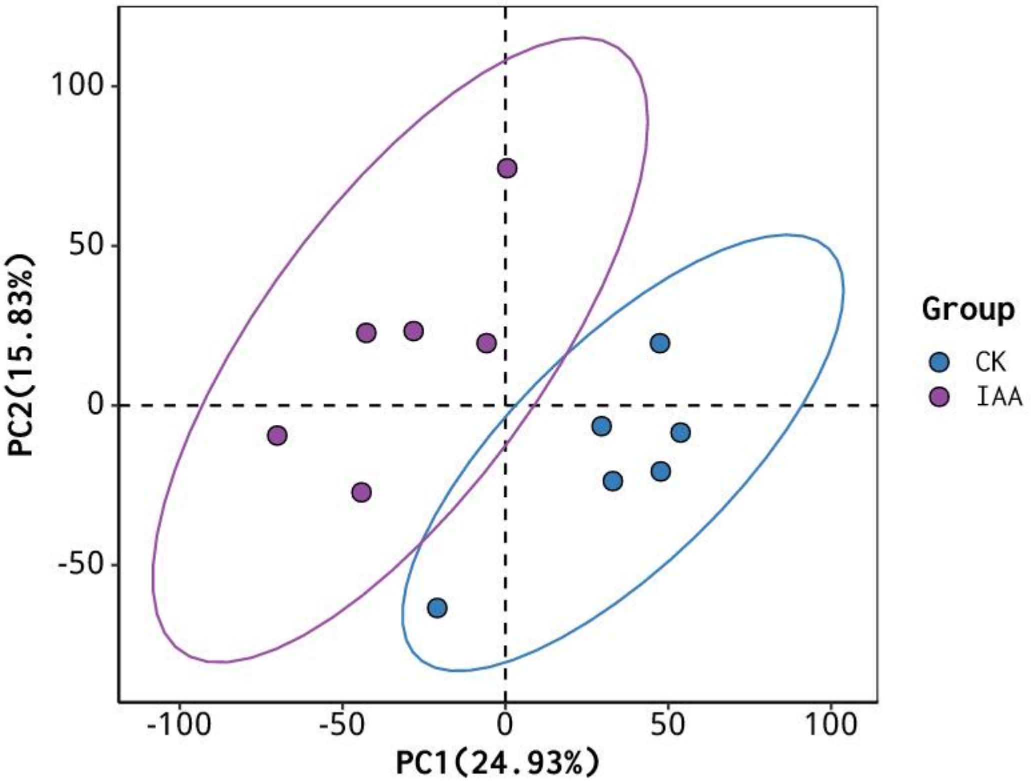

Figure S7. Score chart of PLS-DA analysis model. Horizontal coordinate: PC1 is the principal component 1, the number in parentheses represents the interpretation rate of PC1; Ordinate: PC2 is the principal component 2, the number in parentheses represents the interpretation rate of PC2; Each dot represents a sample, different colors represent different sample groups, and ovals represent 95% confidence intervals

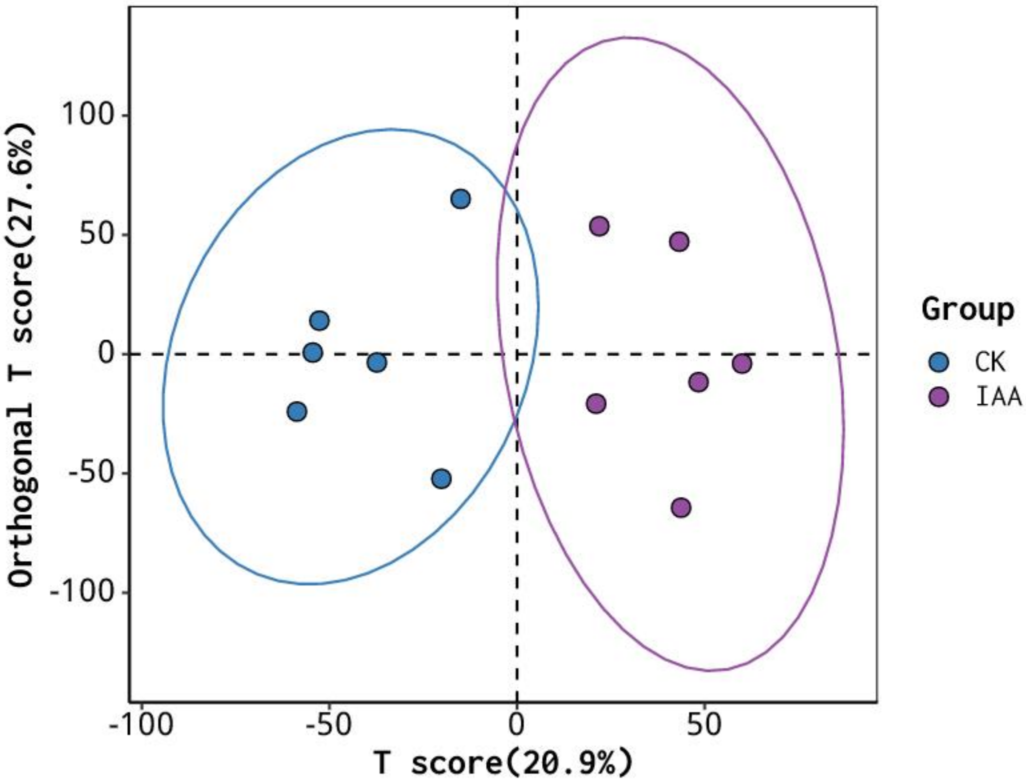

Figure S8. Score chart of OPLS-DA analysis model. In the figure, the horizontal coordinate Tscore represents the predicted principal component score of the first principal component, and the vertical coordinate Orthogonal Tscore represents the orthogonal principal component score. Each dot represents a sample, different colors represent different sample groups, and ovals represent 95% confidence intervals.

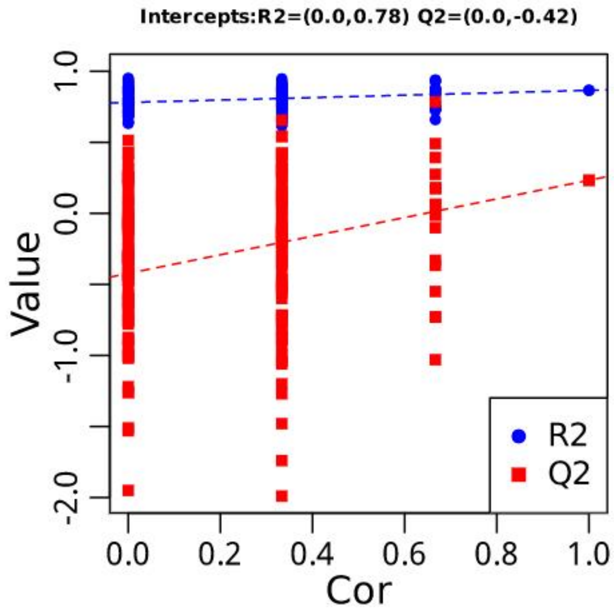

Figure S9.OPLS-DA analysis of model replacement test plots. In this graph, the two dots in the upper right corner represent R2 and Q2 of the actual model. The dots on the left represent the result of the replacement test. Generally, the Q2 obtained from the permutation test needs to be smaller than the Q2 of the model. If you see a red dashed line slanted upward and the intercept of Q2 from the vertical axis is less than 0, the model is good and not overfitted

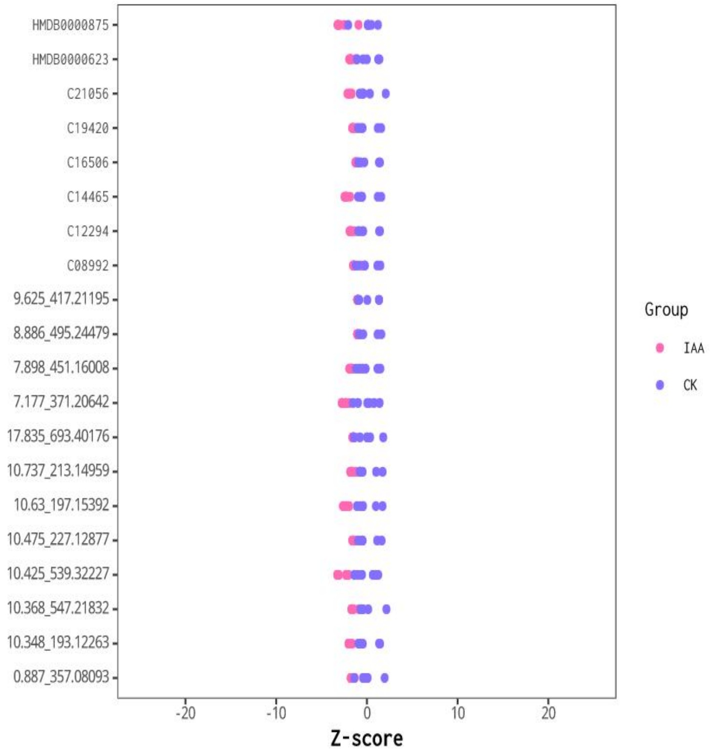

Figure S10.Z-score map of differential metabolites

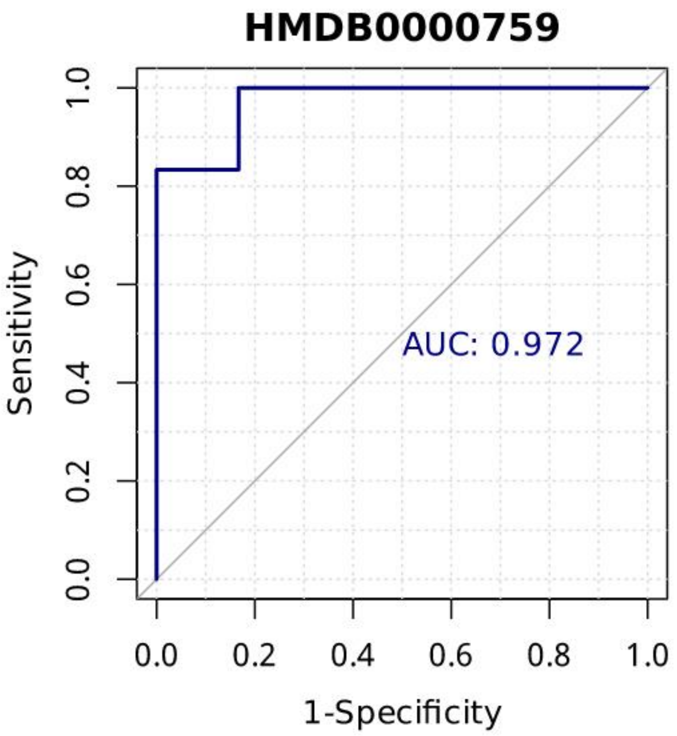

Figure S11.ROC plot. 1-specificity is the horizontal coordinate and sensitivity is the vertical coordinate. The horizontal coordinate is 1-specificity and the vertical coordinate is sensitivity; the area under the line is the AUC value; larger AUC values indicate that the metabolite is more suitable as a biomarker.

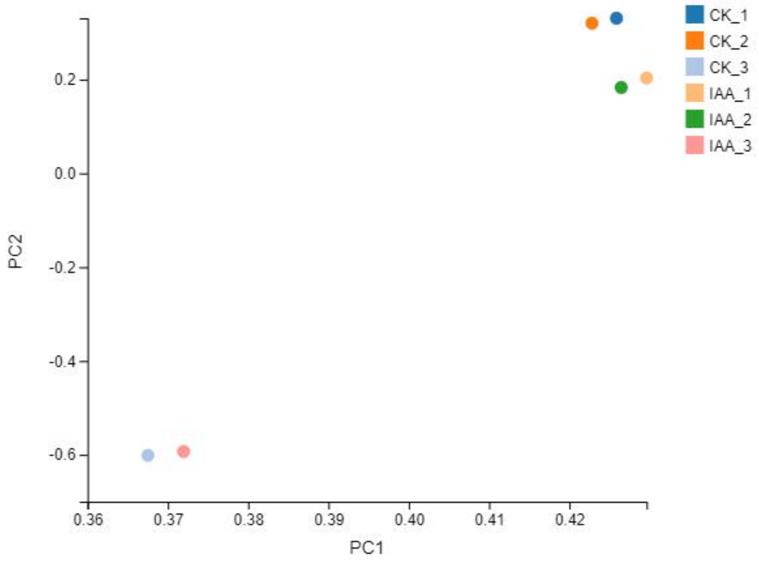

Figure S12. Principal component analysis plot of the transcriptome

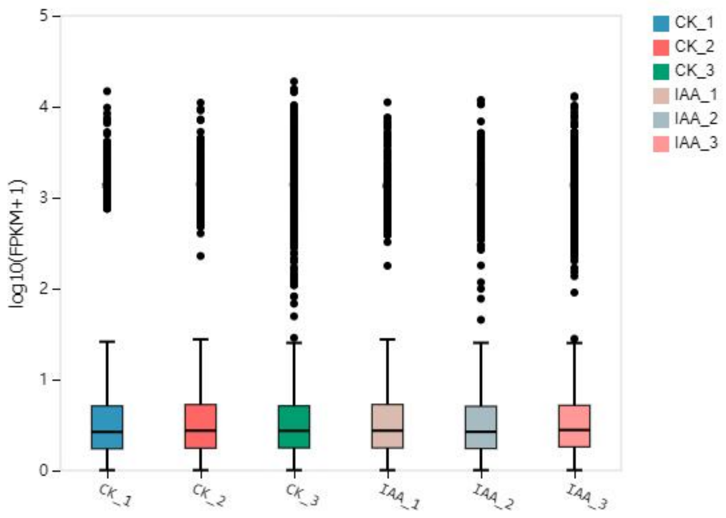

Figure S13. The box plot shows the distribution of gene expression levels of each sample, and the degree of dispersion of data distribution can be observed

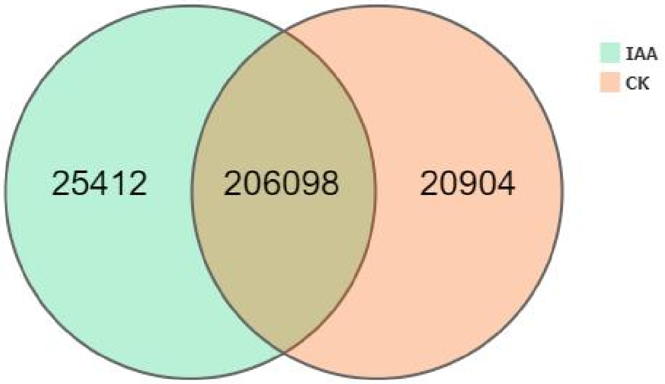

Figure S14. Venn diagram of expression levels between groups

1.2 Supplementary tables

table S1.The volume of each solution added to the test tube

reagent	content（ml）
0.05 mol/L Phosphate buffer	1.5
130 mmol/L Met	0.3
750μmol/L NBT	0.3

100 μmol/L EDTA-Na_2	0.3
20 μmol/L riboflavin	0.3
Enzyme liquor	0.05
H_2O	0.25
Total volume	3.0

Table S2.Coefficient of Variation (CV) evaluation table for QC samples

Num of Total	Num of CV≤30%	Ratio of CV≤30%
10381	9121	0.88

Num of CV ≤ 30% indicates that the reproducibility of the quantitative results for that metabolite is good and can be used for further data analysis. the ratio of the number of metabolites with CV ≤ 30% to the total number of metabolites, Ratio of CV ≥ 0.6, indicates that the assay data of the whole project are satisfactory.
